# Cerebellar Structural and N-Acetylaspartate, Choline, and Creatine Metabolic Profiles in Parkinson’s Disease and Essential Tremor

**DOI:** 10.3390/diagnostics14212430

**Published:** 2024-10-30

**Authors:** Chien-Tai Hong, Cheng-Chang Yang, David Yen-Ting Chen, Shu-Ping Chao, Lung Chan

**Affiliations:** 1 Department of Neurology, Shuang-Ho Hospital, Taipei Medical University, New Taipei 110, Taiwan; ct.hong@tmu.edu.tw (C.-T.H.); junioryang@tmu.edu.tw (C.-C.Y.); 2 Department of Neurology, School of Medicine, College of Medicine, Taipei Medical University, Taipei 110, Taiwan; 3 Taipei Neuroscience Institute, Taipei Medical University, Taipei 110, Taiwan; shh10443@tmu.edu.tw; 4 International Ph.D. Program in Gerontology and Long-Term Care, College of Nursing, Taipei Medical University, Taipei 110, Taiwan; 5 Department of Medical Imaging, Shuang Ho Hospital, Taipei Medical University, New Taipei 235, Taiwan; 6 Department of Radiology, School of Medicine, College of Medicine, Taipei Medical University, Taipei 110, Taiwan

**Keywords:** essential tremor, Parkinson’s disease, magnetic resonance imaging, magnetic resonance spectroscopy

## Abstract

Background: The role of the cerebellum in Parkinson’s disease (PD), particularly in tremor-dominant subtypes, is increasingly recognized. Magnetic resonance imaging (MRI) and magnetic resonance spectroscopy (MRS) provide anatomical and metabolic insights, suggesting compensatory hyperactivity or degenerative changes in the cerebellum in PD. Volumetric analysis of cerebellar structures in MRI images, combined with metabolic profiles from MRS, offers possibilities for differentiating PD from essential tremor (ET). The cerebellum may be a potential therapeutic target due to its role in neurocircuitry of PD and ET. Methods: Brain structural data were obtained using MRI, and cerebellar metabolic profiles, focusing on the quantification of N-acetylaspartate (NAA), choline, and creatine peaks were obtained using MRS. This study enrolled patients with ET and PD, both with and without tremor, as well as disease controls with cerebellar atrophy (including spinocerebellar ataxia and multiple system atrophy). Volumetric analysis of cerebellar structures was performed. Differences in MRI and MRS parameters were analyzed using one-way analysis of covariance with a significance threshold of p < 0.05. Results: From November 2018 to March 2023, 111 patients were enrolled, including 29 ET, 29 cerebellar atrophy, 12 PD without tremor, and 41 PD with tremor. No significant differences in cerebellar volume and N-acetylaspartate/creatine and choline/creatine ratios were found between ET and PD with tremor. Conclusions: This preliminary retrospective study suggests similarities in cerebellar structures and metabolic profiles between ET and PD, highlighting the need for advanced imaging techniques to better differentiate between these conditions. Future research should integrate clinical data, such as tremor severity and cognitive assessments, to explore the relationships with cerebellar MRI parameters.

## 1. Introduction

Tremor is one of the hallmark motor symptoms of Parkinson’s disease (PD) and often serves as an early indicator of the condition. Typically presenting as a “resting tremor,” it most commonly affects the hands, although it can also involve the arms, legs, and jaw. While not all patients experience tremor, it can significantly impact daily activities and quality of life. Effective management of tremor is a key goal in PD treatment, utilizing medications, deep brain stimulation (DBS), or newer technologies like magnetic-resonance-guided focused ultrasound (MRgFUS) [1].

The cerebellum plays a significant, albeit complex, role in the neuropathology of Parkinson’s disease (PD), particularly in relation to PD tremor [2,3]. Traditionally, PD has been primarily associated with degeneration in the substantia nigra and the resultant dopaminergic deficits in the basal ganglia. However, emerging evidence suggests that the cerebellum also contributes notably to the pathophysiology of PD, especially in tremor-dominant forms of the disease. This is thought to be due to dysfunctional connectivity between the cerebellum and the basal ganglia, which are both integral to motor control [4,5]. In PD tremor, the cerebellum may exhibit altered activity patterns, potentially compensating for, or exacerbating, the motor symptoms [6]. Furthermore, the cerebellum’s involvement is supported by the efficacy of cerebellothalamic tract interventions in treating PD tremor [7]. This evolving understanding of the cerebellum’s role in PD underscores the complexity of the disease’s neurocircuitry and highlights the potential for novel therapeutic targets focusing on the cerebellar pathways.

Neuroimaging techniques like magnetic resonance imaging (MRI), functional MRI (fMRI), positron emission tomography, diffusion tensor imaging (DTI), and magnetic resonance spectroscopy (MRS) have been employed to assess the cerebellar activity and connectivity in PD patients, particularly those with tremor-dominant symptoms [8,9]. On one hand, several studies using these imaging modalities have revealed increased cerebellar activation in PD patients, suggesting a compensatory response to the dopaminergic loss in the basal ganglia. This hyperactivity is hypothesized to be part of an adaptive mechanism to counterbalance the motor deficits associated with PD [10,11]. Additionally, some studies have found altered connectivity between the cerebellum and other regions implicated in motor control, such as the motor cortex and basal ganglia, further supporting the cerebellum’s active role in the disease’s pathophysiology [12,13,14]. Conversely, other studies have reported reduced cerebellar activity or atypical cerebellar responses in PD patients. These findings imply a potential degenerative process or functional disconnection within the cerebellum itself or in its connections with the basal ganglia and cortical areas [15]. The discrepancies in these findings may be attributed to various factors such as differences in study design, patient selection (including stages of PD and presence of tremor), and the specific neuroimaging techniques and analytical methods used.

Essential tremor (ET) is another prevalent tremor disorder. ET is the most common movement disorder, characterized by a postural or kinetic tremor, primarily affecting the hands and forearms, though it may also involve the head, voice, and other body parts. Unlike PD, where tremor typically occurs at rest, the tremor in ET is most pronounced during voluntary movement or when maintaining a posture. The exact pathophysiology of ET remains unclear, but it is thought to involve abnormal activity in the cerebellum and its associated pathways. ET is often familial, with a hereditary pattern seen in up to 50% of cases, and its prevalence increases with age. Clinically, ET can range from mild and non-disabling to severe, significantly impairing daily functions such as eating, writing, or speaking. While treatment options such as beta-blockers and anti-seizure medications may provide symptomatic relief, surgical interventions like DBS or MRgFUS are considered for cases that are refractory to medical therapy [16]. Clinically, segregating ET with PD tremor is challenging [17]. Differentiating PD tremor from ET using cerebellar MRI is not always conclusive. Conventional structural MRI, including cerebellar imaging, is typically normal in both PD and ET. However, some studies suggest subtle differences in the cerebellum and related structures. For instance, in ET, there may be changes in cerebellar volume or integrity [17]. Advanced MRI techniques, such as fMRI, DTI, and MRS, can provide more detailed insights into brain function and connectivity [18]. These methods have shown some differences in cerebellar activity and connectivity patterns between PD and ET. For example, altered cerebello–thalamo–cortical pathways may be more characteristic of ET, while changes in the basal-ganglia–cortical circuits are more typical of PD [19]. However, these findings are not consistent across all studies and are not specific enough to definitively differentiate ET from PD.

Proton MRS can provide robust metabolic profiles, such as N-acetylaspartate (NAA), choline (Cho), and creatine (Cr) peaks, in the brain in the clinical setting. NAA is a marker of neuronal function, with decreased levels reported in neurodegenerative diseases, while creatine serves as a stable reference [20,21]. Previous research has shown that MRS can aid in diagnosing and monitoring PD, where lower NAA-to-Cr ratios are linked to cognitive impairment and affected brain regions [22,23]. In ET, a reduced NAA-to-Cr ratio in the cerebellum correlates with tremor severity, suggesting neuronal damage [24]. Therefore, we aim to determine whether NAA, Cho, and Cr peaks in the cerebellum show distinct patterns in PD and ET, providing insights into their metabolic differences.

The use of artificial intelligence (AI) in volumetric analysis of brain MRI has gained considerable popularity [25,26], offering several significant advantages. AI algorithms provide increased accuracy and consistency in analyzing complex brain imaging data, surpassing manual methods, which are prone to observer variability. AI-based volumetric analysis of the cerebellum has been applied in the research field for more than a decade and has obtained fruitful results [27,28,29,30].

Given the clinical challenges and the urgent need to differentiate essential tremor (ET) from Parkinson’s disease (PD), this study was conducted in light of the growing understanding of the cerebellum’s pathological role in both diseases. It focuses on exploring the potential of using cerebellar MRI volumetric parameters in conjunction with magnetic resonance spectroscopy (MRS) to distinguish between ET and PD, leveraging the advancements in AI-based brain MRI volumetric analysis.

## 2. Materials and Methods

### 2.1. Patient Selection

This retrospective study was approved by the Joint Institutional Review Board of Taipei Medical University (Approval No. N202311040). Medical records from Shuang Ho Hospital, a university-affiliated hospital, were reviewed between Nov. 2018 and Mar. 2023. Within this period, 111 patients with early stage PD, separated into with/without tremor and laterality of tremor, and ET who had undergone MRI and cerebellar MRS were enrolled. We also enrolled patients with cerebellar atrophy (spinocerebellar ataxia (SCA) or multiple system atrophy (MSA)) as the disease control. PD was diagnosed on the basis of the UK PD Brain Bank diagnostic criteria [31]. The present study collected baseline demographic data of the participants, including age, sex, and the symptoms of PD, especially the laterality of tremor.

### 2.2. MRI Protocol

**Figure 1:**
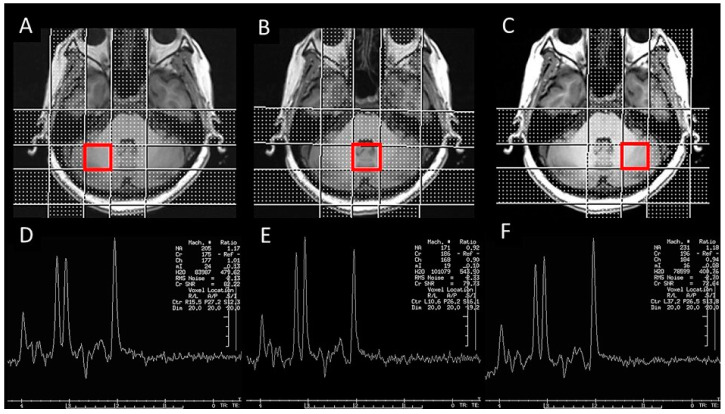
A representative schematic of cerebellar ROIs for MR spectroscopy. (**A**–**C**) Right cerebellar hemisphere, cerebellar vermis, and left cerebellar hemisphere ROIs. (**D**–**F**) Corresponding spectra for each ROI.

Imaging was performed using a 3T (Discovery MR750; GE Healthcare, WI, USA) MRI scanner with an 8-channel head coil. High-resolution T1-weighted cerebellar structural images were acquired using a 3D fast spoiled gradient-echo sequence (BRAVO), with the following parameters: repetition time (TR) = 8.2 ms, echo time (TE) = 3.2 ms, inversion time (TI) = 450 ms, flip angle = 12°, field of view = 256 mm, 172 slices, resolution = 1 × 1 × 1 mm^3^, and axial acquisition. Cerebellar MRS data were acquired using a volume-selective, inversion-recovery, water-suppressed, spectroscopy sequence (PROBE), with the following parameters: TR = 1500 ms, TE = 144 ms, spectral bandwidth = 2000 Hz, sampling resolution = 20 × 20 × 20 mm^3^, and number of excitations = 128. Prior to each acquisition, automatic shimming and water suppression were performed. Three voxels of interest were positioned, each in the right cerebellar hemisphere, left cerebellar hemisphere, and cerebellar vermis (Figure 1).

### 2.3. Quantitative Volumetric Analysis

**Figure 2:**
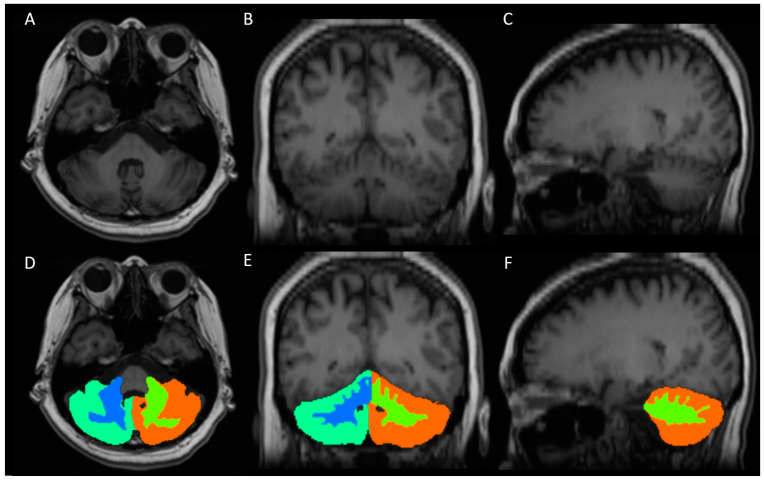
A representative case of cerebellar segmentation. (**A**–**C**) Axial, coronal, and sagittal view of T1-weighted high-resolution image, respectively. (**D**–**F**) Axial, coronal, and sagittal view of right cerebellar gray matter mask (cyan), right cerebellar white matter mask (blue), left cerebellar gray matter mask (orange), and left cerebellar white matter mask (green), respectively.

Quantitative volumetric analysis was performed using FreeSurfer software (version 7.1.0), which is an established and widely used approach for brain segmentation [32]. The recon-all pipeline, which involves intensity normalization, skull-stripping, motion correction, affine registration to the template, and probabilistic atlas-based segmentation, was employed for subcortical segmentation and to acquire left and right cerebellar gray and white matter volumes and whole-brain volume (Figure 2). One neuroradiologist (D.Y.-T.C.) reviewed the automatic subcortical segmentation to ensure alignment with the intended anatomical regions. Subjects with significant deviations in segmentation or inconsistencies between the volume of interest in the spectral study were excluded from the analysis. During the volumetric analysis of the brain using FreeSurfer, substantial variation in head and brain size, including the cerebellum, was observed between the individuals, particularly between males and females. To account for this variation, we normalized the volumes of cerebellar gray and white matter by dividing them by the total brain volume, which was also obtained using FreeSurfer.

### 2.4. MRS Quantitative Analysis

MRS raw data were analyzed using vendor-provided software (GE SAGE 7). A neuroradiologist qualitatively evaluated each spectrum to ensure the presence of NAA, Cr, and Cho peaks. NAA/Cr and Cho/Cr concentration ratios were recorded.

### 2.5. Statistical Analyses

All statistical analyses were performed using SPSS for Windows 10 (version 26.0; SPSS, Chicago, IL, USA). Continuous variables are presented as mean ± standard deviation, and categorical variables are presented as percentages with corresponding 95% confidence intervals. The differences in the cerebellar volume and cerebellar MRS parameters were analyzed using one-way analysis of covariance (ANCOVA) with Bonferroni-adjusted post hoc tests, with adjustment for age and sex. ANCOVA is a statistical technique that combines the features of Analysis of Variance (ANOVA) and regression. ANCOVA is primarily used to compare the means of different groups while controlling for one or more covariates (confounding variables) that may influence the outcome variable. This adjustment helps to isolate the effect of the independent variable by reducing the influence of confounding factors, leading to more accurate comparisons between groups. A *p*-value of <0.05 was considered statistically significant.

## 3. Results

From November 2018 to March 2023, this study included 111 patients who underwent brain MRI. Among the included patients, 29 had ET, 29 had cerebellar atrophy (either SCA or MSA), 12 had PD without tremor, and 41 had PD with tremor (right-side tremor was prominent in 22 patients). Significant differences were observed in the sex distribution across patient groups. The patients with ET and patients with SCA/MSA were younger than the patients with PD (Table 1).

**Figure 3:**
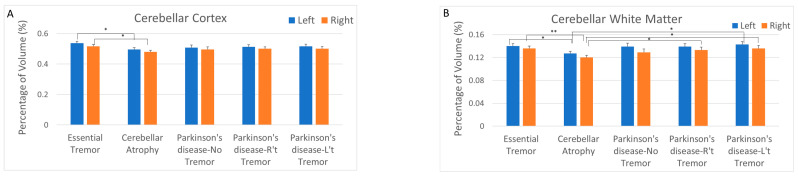
Comparison of cerebellar volume, including cortex (**A**) and white matter (**B**), between different groups. * *p* < 0.05; ** *p* < 0.01.

A quantitative volumetric analysis was performed on all brain MRIs. The quality of the subcortical segmentation with FreeSurfer was approved by the neuroradiologist in all cases. This analysis identified and quantified the right and left cerebellar gray and white matter, with adjustment for the volume of each area based on the overall size of the brain. Notably, a significant difference in the cerebellar cortical volume was found only between the patients with ET and cerebellar atrophy and the patients with PD. This difference was not observed between the patients with ET and the patients with PD, irrespective of the presence of tremor. No discernible difference was noted in the cerebellar volume between the patients with PD with and without tremor. Furthermore, in patients with PD with tremor, tremor laterality was not correlated with the differences in the left or right cerebellar volume (Figure 3A). Regarding the cerebellar white matter, similar findings were obtained; moreover, the patients with cerebellar atrophy exhibited a significantly reduced cerebellar white matter volume compared with all of the other patients. However, no significant difference was observed in the cerebellar white matter volume between the other four patient groups (Figure 3B).

**Figure 4:**
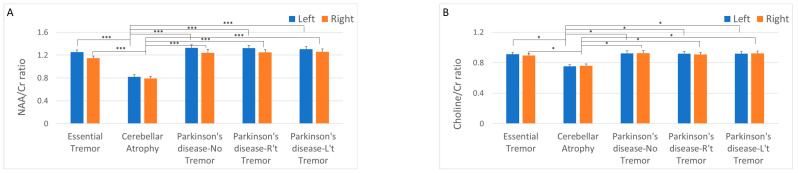
Comparison of cerebellar magnetic resonance spectroscopy parameters between different groups. (**A**) N-acetylaspartate (NAA) to creatine (Cr) ratio, which indicates neuronal integrity. (**B**) Choline (Cho) to Cr ratio, which indicates metabolism. * *p* < 0.05; *** *p* < 0.01.

In addition to the volumetric analysis of cerebellar structures in MRI images, cerebellar metabolic profiles were investigated using MRS. Two MRS parameters were applied: NAA/Cr ratio and Cho/Cr ratio. NAA is a compound found in neurons that is considered a marker of neuronal health and density. Cr serves as a marker of cellular energy metabolism. The NAA/Cr ratio is used to assess neuronal integrity and function. Cho is involved in cell membrane metabolism. Elevated levels of Cho are often associated with increased cell membrane turnover, which is common in conditions such as cancer or inflammation [33]. In the present study, the differences in the MRS parameters between the groups were consistent with those in structural MRI. Both the NAA/Cr and Cho/Cr ratios were significantly lower in the cerebellar atrophy group than in the other four groups. However, no significant differences were observed in the ratios between the other four patient groups (Figure 4A,B).

## 4. Discussion

The current study demonstrated that AI-assisted volumetric analysis detected no significant differences in cerebellar volume between individuals with ET and PD with tremor. Additionally, MRS revealed no notable disparities in NAA/Cr and Cho/Cr ratios between the ET and PD tremor groups. When compared to individuals with cerebellar atrophy resulting from SCA or MSA, the cerebellar structural and metabolic profiles of patients with ET and PD showed markedly less impairment. Consequently, differentiating between these two diseases based on cerebellar MRI proved to be impractical.

Differentiating between ET and PD tremor in clinical settings poses significant challenges, often leading to a high frequency of misdiagnosis. Clinically, both conditions present with tremor, but the nature and timing of these tremors are distinct—ET primarily involves action tremor, worsening with movement, while PD is characterized by resting tremor. However, in early stages or atypical presentations, these differences can be subtle, complicating accurate diagnosis. Furthermore, some patients with PD may initially present with symptoms resembling ET, or vice versa, leading to further confusion [17]. However, differentiating between PD tremor and ET is crucial for determining the most appropriate treatment approach. PD tremor is typically a resting tremor that responds well to dopaminergic medications, such as levodopa, which can help to improve overall motor symptoms in PD patients. In contrast, ET is primarily a kinetic or postural tremor, which does not respond to dopaminergic therapy but may be managed with medications like beta-blockers (e.g., propranolol) or anticonvulsants (e.g., primidone) [16,33]. Furthermore, surgical options, such as DBS, target different brain regions in each condition, as follows: the subthalamic nucleus or globus pallidus for PD, and the thalamus for ET [34]. Differentiating between tremor-dominant PD and ET using structural MRI poses challenges. Purrer et al. employed AI-based analysis, primarily focusing on the cerebral and basal ganglia regions while paying minimal attention to the cerebellum. Their findings indicated a similarity in structural changes between these two diseases [25]. Given the more substantial evidence of cerebellar pathology in ET, the current study also sought to utilize AI-based structural MRI and MRS to distinguish between these conditions. This approach included a more detailed examination of the cerebellum by separating its cortex and white matter, as well as considering cerebellar laterality. Unfortunately, neither cerebellar white/gray matter volume nor the MRS of cerebellar hemisphere demonstrate the difference between PD with tremor and ET. Compared with the disease control in this study, the structural MRI and MRS from patients with SCA and MSA, both ET and PD significantly differed from them. Our results revealed a failure to distinguish ET from PD through the structural MRI and MRS of the cerebellum, and this may be because these methods primarily assess macroscopic structures and metabolic profiles, which may not capture the subtle cellular or functional changes that differentiate ET and PD. Previous research has identified cerebellar voxel-based morphometry (VBM) differences between age-matched PD and ET [35]. Although age was considered in our analyses, this negative finding may also imply heterogeneous age-related structural shrinkage across cerebellar regions [36]. Advanced imaging techniques, such as quantitative susceptibility mapping (QSM), have shown promise in detecting iron accumulation and its association with motor and cognitive impairments in PD [37,38]. These advanced modalities that focus on cellular activity or neuronal connectivity may be more appropriate for distinguishing between ET and PD in future studies.

The heterogeneity of PD motor subtypes, particularly those with and without tremor, manifests in several aspects. The tremor-dominant subtype is characterized by the prominence of resting tremor with relatively milder bradykinesia and rigidity, while the non-tremor subtype involves marked bradykinesia (slowness of movement) and rigidity, with less prominent or absent tremor. These subtypes not only differ in motor symptom presentation, but may also show variations in disease progression, response to treatment, and underlying pathophysiology. Patients with PD without tremor, especially those exhibiting a postural instability and gait difficulty (PIGD) phenotype, often experience more pronounced non-motor symptoms, such as cognitive impairment, sleep disturbances, and autonomic dysfunction earlier in the disease course, compared to their counterparts with tremor. Additionally, the rate and pattern of disease progression vary between these PD subtypes. Typically, patients with PD with tremor exhibit a slower progression of motor symptoms and a more favorable overall prognosis. In contrast, PD patients without tremor, particularly those with PIGD features, tend to experience a more rapid progression, marked by an earlier onset of disability, cognitive decline, and other complications [39,40,41,42,43]. Despite these clinical differences, neuroimaging has not been definitively established as a tool for differentiating between these subtypes [44]. Considering the potential involvement of cerebellar neuropathology in PD, such as compensating for motor deficits caused by the loss of dopaminergic neurons or the development of abnormal activity due to changes in neural circuits as the brain adapts to dopamine loss, it has been hypothesized that cerebellar neuroimaging might play a distinct role in identifying PD tremor [45,46,47,48]. However, this study has demonstrated no significant structural differences in the cerebellum between PD patients with and without tremor. Additionally, for PD patients with unilateral tremor, no disparity in cerebellar cortical and white matter volume was observed between the ipsilateral and contralateral sides.

MRS is known to detect changes in the concentration of brain metabolites or chemicals, which are often the result of alterations in cellular metabolism and can precede structural changes. MRS may identify metabolic changes indicative of early disease stages in various neurological conditions, including neurodegenerative diseases like Alzheimer’s disease, before MRI reveals significant structural loss [49]. This property renders MRS a potentially valuable tool for early diagnosis and disease progression monitoring. However, this study also applied MRS to assess the neuronal metabolites in the bilateral cerebellar hemispheres and found no differences between PD patients with and without tremor, as well as between the ipsilateral and contralateral sides in PD with tremor. These findings from structural MRI and MRS suggest that cerebellar neuroimaging might not be capable of differentiating PD with and without tremor, despite their distinct clinical manifestations and prognoses. This may also call into question the neuropathological role of the cerebellum in PD tremor. Research has indicated that the origin of PD tremor primarily stems from basal ganglia dopaminergic degeneration, with the cerebellum serving merely as a modulator [5].

The principal strength of this study lies in the comprehensive assessment of the cerebellum, employing both structural MRI and MRS to evaluate cellular metabolites. This dual approach is particularly advantageous in neurodegenerative diseases, where structural changes may be delayed. Additionally, the utilization of AI-based FreeSurfer for cerebellar volumetric analysis mitigates the bias inherent in manual assessments, ensuring uniformity in the standard of analysis. FreeSurfer provides an automated, standardized pipeline for brain volume segmentation, allowing for highly reproducible and accurate measurements across different subjects and time points. This automated approach reduces human error and variability that can arise from the manual delineation of brain structures, particularly in complex regions like the cerebellum. Furthermore, FreeSurfer’s ability to perform cortical and subcortical segmentation based on probabilistic atlases enhances the precision of volumetric analysis, leading to more reliable cross-study comparisons. The software’s widespread use and validation in neuroimaging research add further credibility, allowing for comparability with other studies and facilitating large-scale data integration. These advantages make FreeSurfer an essential tool for ensuring consistency, objectivity, and accuracy in cerebellar volumetric analyses, improving the overall quality of neuroimaging studies. Lastly, the inclusion of a disease control group, comprising patients with significant cerebellar atrophy, served as a benchmark for comparing cerebellar volume and cellular metabolite levels. Our findings indicate that neither ET nor PD exhibit extensive cerebellar impairment. Thus, future research into the pathogenic role of the cerebellum in tremor disorders should focus more on dysfunction rather than on degeneration.

The current study is subject to several limitations. Firstly, as a retrospective analysis, it lacks a healthy control group, featuring only a disease control group. This absence limits the scope for making definitive conclusions about the normality of cerebellar volume and MRS parameters in PD patients, especially given the region-specific aging processes in the cerebellum observed in healthy adults [31]. Secondly, the marked differences in age and disease duration between the PD and ET groups introduce an element of bias. While one-way ANCOVA, when adjusted for age and sex, may partially mitigate this bias, it does not fully address the potential impact of these disparities. Another relevant consideration is the use of GE SAGE 7 software for MRS analysis in the current study. While this software is widely used in clinical settings, employing specialized software such as LCModel (Version 6.3), jMRUI (Version 7.0), or Osprey (Version 2.8.0), which are considered the gold standards for spectral analysis, may provide more refined and accurate results. Future studies could benefit from integrating these specialized tools to enhance the precision of the spectral analysis. Thirdly, given that recent studies have focused on the quantitative alteration of specific metabolites, such as Cr, rather than conventional ratios [50], it may be valuable to consider absolute changes in future prospective studies. Lastly, this study does not include a detailed assessment of the motor symptoms of PD and ET, nor does it include cognitive measurements, as cognitive assessment is not routinely conducted in clinical practice for these conditions. This omission precludes a more thorough investigation into the relationship between tremor severity, cognitive functioning, and cerebellar MRI and MRS parameters, which could have provided deeper insights into the pathophysiology of these tremor disorders.

## 5. Conclusions

Our findings suggest certain similarities in cerebellar structure and NAA to Cr ratio profile between ET and PD, though the differentiation of these conditions using conventional MRI techniques remains challenging. Further studies employing advanced neuroimaging methods, such as DTI, QSM, or specialized MRS sequences, are necessary to reach more definitive conclusions. This study also prompts further investigation into the cerebellum’s pathogenic role in PD tremor disorders, suggesting a possible focus on dysfunction rather than on degeneration. Future research should incorporate additional clinical data, such as tremor severity and cerebellar cognitive assessments, to explore the relationships with various cerebellar MRI parameters.

## Figures and Tables

**Table 1:** Participant demographic information.

	ET	Cerebellar Atrophy (SCA or MSA)	PD Without Tremor	PD with Tremor, Right Side-Predominant	PD with Tremor, Left Side-Predominant
Gender ratio (F/M)	16/13	16/13	6/6	10/12	11/8
Age, mean (SD)	51.38 (20.07)	53.34 (14.08)	69.08 (4.62)	68.54 (7.03)	66.79 (8.16)

Abbreviations: ET, essential tremor; MSA, multiple system atrophy; PD, Parkinson’s disease; SCA, spinocerebellar ataxia.

## Data Availability

Please contact the corresponding author (L. Chan). The availability of data and materials requires permission from the Joint Institutional Review Board of Taipei Medical University.
